# The effect of experience in movement coordination with music on polyrhythmic production: Comparison between artistic swimmers and water polo players during eggbeater kick performance

**DOI:** 10.1371/journal.pone.0238197

**Published:** 2020-08-25

**Authors:** Ravisara Vathagavorakul, Tomohiro Gonjo, Miwako Homma

**Affiliations:** 1 Graduate School of Comprehensive Human Sciences, University of Tsukuba, Tsukuba, Japan; 2 Department of Physical Performance, Norwegian School of Sport Sciences, Oslo, Norway; 3 Faculty of Health and Sport Sciences, University of Tsukuba, Tsukuba, Japan; University of Pittsburgh, UNITED STATES

## Abstract

The aim of this study was to compare artistic swimmers (ASs) and water polo players (WPs) in their polyrhythmic production ability and entrainment between arm and leg motion frequency. Nine ASs and nine WPs participated in the study. First, we assessed the natural eggbeater kick frequency of each participant without any additional motion for 20 s. We then required the participants to perform a circular arm movement in synchronization with two sequences of metronome rhythms (either 100%, 80% and 120% or100%, 120% and 80% of their natural eggbeater kick frequency) while maintaining their natural eggbeater kick frequency. All tasks were repeated three times. The participants’ performances were recorded by a motion capture system synchronized with the metronome. A two-way mixed-design ANOVA was performed on the coefficient of variation of natural eggbeater kick frequency obtained from the first task to confirm the consistency of participants’ kicking motion. In the second task, a three-way mixed-design ANOVA was performed on the average frequency of the arm and leg motions to assess the entrainment between the two. In the first task, there were no significant main effects and interaction between group and trial in the coefficient of variation of eggbeater kick frequency, suggesting that both WPs and ASs maintained their natural eggbeater kick frequency equally consistently. In the second task, however, WPs were not able to maintain their natural eggbeater kick frequency when they were required to do circular arm movements at 120% tempo (*p* < .01). On the other hand, ASs successfully maintained their natural eggbeater kick frequency with all metronome rhythms, suggesting that they have a better polyrhythmic production ability than WPs.

## Introduction

Coordination of human movements between the upper and lower limbs can produce a variety of temporal and spatial combinations Swinnen [1]. The quality of this coordination depends on the direction and frequency of the arm and leg movements [2]. Kelso [3] showed that inter-limb behavior in humans is characterized by two coordination modes: in-phase and anti-phase. During in-phase limb coordination, two limbs move in synchrony with no phase lag between them, whereas anti-phase limb coordination has a phase shift between limb motions of exactly 180 degrees. Phase differences of more than zero but less than 180 degrees are referred to as out-of-phase movement. Wannier, Bastiaanse, Colombo and Dietz [4] demonstrated that the coordination between upper and lower extremities in cyclic human movements, such as walking and swimming, was frequency locked (i.e. temporally associated), and was preserved in a harmonic manner. Their results indicated that the relationship between arm and leg movements tends to remain either in-phase or anti-phase, due to the difficulty involved in changing one’s coordination from anti-phase to in-phase or vice versa. In-phase movements require bilateral homologous muscle groups to contract synchronously while they are contracted in an alternating fashion during anti-phase movements [5]. Studies have also shown that in-phase movements are more accurate and require less attention than anti-phase movements in movements such as finger tapping, cyclical bimanual movement, or upper and lower limb movements [1, 6–9]. Coordination between upper and lower limbs is more stable and easier to perform in an in-phase than in an anti-phase manner [1, 2, 10, 11], and people tend to change their inter-limb coordination pattern from anti-phase to in-phase, since anti-phase movements are unstable and difficult to maintain [4, 12].

The process of synchronizing multiple movements with each other involves an interaction between independent rhythmic systems, which is called entrainment [13]. In human movements, entrainment can be induced by external stimuli (which can be auditory or visual cues, such as music or another person’s movements) or by internal movements of body parts. For example, when a musician is playing a musical instrument, i.e., producing rhythmical sequences, the musician can adapt the music to their bodily rhythms such as breathing cycles. On the other hand, the bodily rhythms can also be adapted to a chosen musical rhythm by, for example, changing the breathing rhythm [14]. In other words, musicians utilize the interaction between the one bodily rhythm (such as finger movements) to another (e.g., breathing cycle). Several studies [14–17] have shown that once entrainment happens, phase relationships amongst different limb movements shift from out-of-phase coordination to relatively fixed in-phase or anti-phase movements. Moreover, as the frequency of motion increases, in-phase movements become more stable and require less attention than either anti-phase or out-of-phase movements.

Although it is easier for limbs to function with in-phase coordination due to this entrainment process, some advanced, highly complex tasks require the limbs to move independently from each other. For example, achieving high-level performance in music, dance or sports entails mastering and producing an exquisite combination of in-phase, anti-phase and out-of-phase limb movements [18]. In many such performances, polyrhythmic production (i.e. limbs moving in different frequency) is essential for quality performance [17]. Studies have shown that highly skilled musicians can perform polyrhythmic tasks better than non-musicians and that polyrhythmic ability can be acquired through extensive training [19–24].

In some contexts, using complex combinations of phase relationships can optimize energy expenditure and mechanical advantage so that one can complete the intended tasks while minimizing effort. Artistic swimming and water polo are two such contexts. Both disciplines require athletes to perform two very different categories of motor sequences at once, one above and one under the water. Therefore, these athletes are accustomed to complex inter-limb coordination and polyrhythmic production. Vathagavorakul, Gonjo and Homma [25] studied polyrhythmic production, in the form of finger and foot tapping, by artistic swimmers (ASs) and water polo players (WPs), demonstrating that the two groups had different polyrhythmic abilities.

The eggbeater kick (EBK) is a fundamental skill in both water polo and artistic swimming. It is a complex anti-phase movement that requires both legs kick at frequencies that can generate enough lift forces to keep the body above the water [26]. Homma and Homma [27] showed that ASs performed EBKs during about 40% of their total performance routines, whereas Smith found that WPs performed EBKs for more than 55% of the duration of a game [28]. ASs must perform above-water movements in synchronization with music, the tempo of which frequently changes in the course of a performance routine while maintaining their optimal EBK frequency. ASs must engage in polyrhythmic production using both their upper and lower extremities. Similarly, WPs perform the EBK to provide a stable platform for passes, shots, or defensive moves. These performances also include fake shots, throws, or hand waving during the defense. These performances, thus, involve short rhythmic movements of their upper extremities that are not in the same frequency as their EBK frequency. Thus, WPs also engage in polyrhythmic production between their upper and lower extremities, but without any need to synchronize their movements with music.

The presence of absence of music can affect the process of skill development. Several studies have compared the movement coordination skills of musicians and non-musicians, finding that musicians had better inter-manual coordination and polyrhythmic production but less entrainment than non-musicians [21, 29, 30]. However, few researchers have studied these abilities in athletes. It has been reported that ASs have better polyrhythmic production ability than WPs and perform at a comparable level to musicians on tapping tasks [25]. However, it is unclear whether ASs also outperform WPs on more complex tasks that both groups are regularly required to perform, such as eggbeater kicks. Since the movements performed by ASs and WPs exhibit both similarities (complex upper and lower limb coordination in the water) and differences (sound cues for ASs but not for WPs), comparing the performances of these two groups of athletes should provide insight into how experiences in coordinating movements with external sounds affect polyrhythmic performance in sports. Understanding the effect of music or sound stimuli on the movement will be beneficial for ASs and WPs, and potentially other athletes, to consider how to improve polyrhythmic production and, thus, inter limb coordination to effectively produce required motor tasks.

Accordingly, the objective of the present study was to compare polyrhythmic production ability and entrainment between ASs and WPs during eggbeater kicking tasks. Since ASs are required to match their upper extremity performance to a wide range of music tempos, it was hypothesized that ASs would display less entrainment movement patterns than WPs.

## Methods

### Participants

Two groups of well-trained participants (ASs and WPs) volunteered to participate in this study. The ASs (nine females; age 21.8±4.40 years, height 163 ± 4.00 cm, body mass 53.9 ± 4.86 kg) had participated in international and national competitions and averaged 12.6 ± 3.40 years of artistic swimming experience. The WPs (nine males; mean age 22.0 ±3.39 years, height 172 ± 6.76 cm, body mass 74.9 ± 10.7 kg) had 10.6 ± 2.29 years of water polo experience. Both groups, on average, had less than two years of experience in learning singing or a musical instrument (WPs, 1.10 ± 1.36 years; ASs, 1.45 ±3.36 years). We recruited the participants from February to March 2019. Inclusion criteria included 18 years of age or older, elite artistic swimmers or water polo players defined as athletes competing at the international or national level, and being able to complete eggbeater kick without subjective fatigue or notable change in motion for one minute. Exclusion criteria included being diagnosed with cardiovascular disease, injured, and having any language or cognitive barrier that prevented them from following instructions. The testing procedures were approved by the University of Tsukuba Ethics Committee, and each participant signed an informed consent form.

### Experimental tasks

The participants were instructed to perform two tasks. The purpose of the first task was to determine the natural EBK frequency—defined as the frequency at which participants could most comfortably maintain their body height (upper edge of sternum level) above the water while performing EBKs at their own pace—for each participant. Thus, the first task required only leg movements. In contrast, in the second task, participants were asked to perform EBKs together with arm movements in synchronization with metronome beats.

#### Task 1: Determining natural EBK frequency

The participants were instructed to perform EBKs at their own pace while maintaining the upper edge of their sternum above the water. Moreover, they were asked to hold their entire dominant arm above the water while their other hand was placed on the hip. The participants were strictly forbidden to do sculling. They were asked to complete three EBK trials of 20 s each, and the last 15 s of each trial were analyzed to determine the natural leg frequency.

#### Task 2: Arm and leg coordination with metronome beats

**Fig 1:**
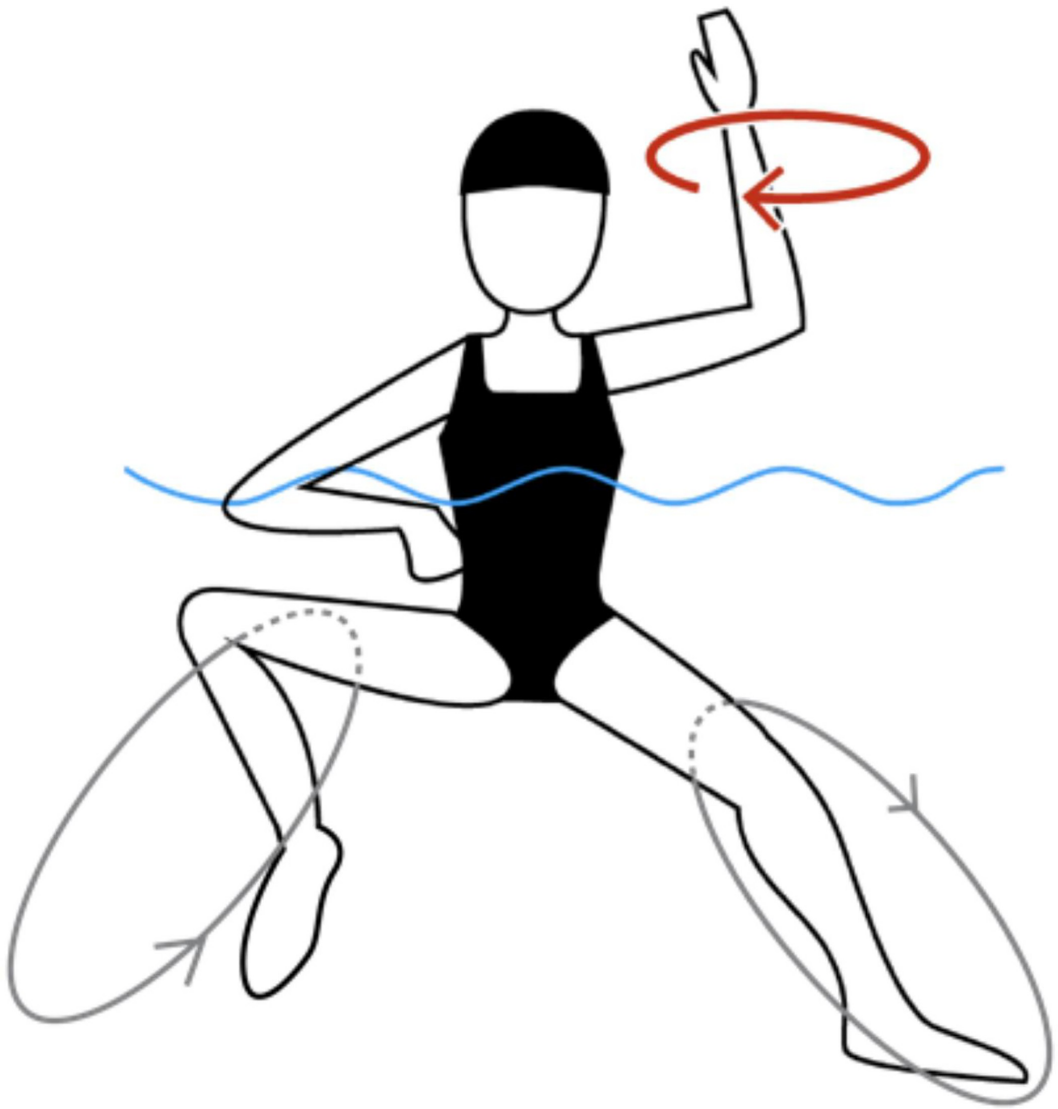
Combined arm and leg actions during task 2, arm and leg coordination with metronome beats.

In the second task, the participants were instructed to perform EBKs while moving one arm in a circular motion above the water at the same time. The circular arm movements (CAMs) were to be synchronized with metronome beats while the participants maintained EBK movements with the legs at their natural frequency (Fig 1). All participants were instructed to perform CAMs using the elbow as a pivot point and drawing a circle with their hand as wide as possible. We varied the metronome frequency between three tempos: slow, normal and fast (defined as 80%, 100% and 120% of natural EBK frequency, respectively). The three frequencies were combined to make one set of movements. To minimize the effect of the task order on performance, we used two different sequences: normal-slow-fast and normal-fast-slow. In both cases, we started with the natural EBK frequency to confirm that the participants could perform EBKs together with CAMs at this frequency. Had any participants been unable to perform this entrainment, we would have corrected them and asked them to start over. However, all participants could synchronize CAMs with EBKs at their natural frequency.

**Fig 2:**
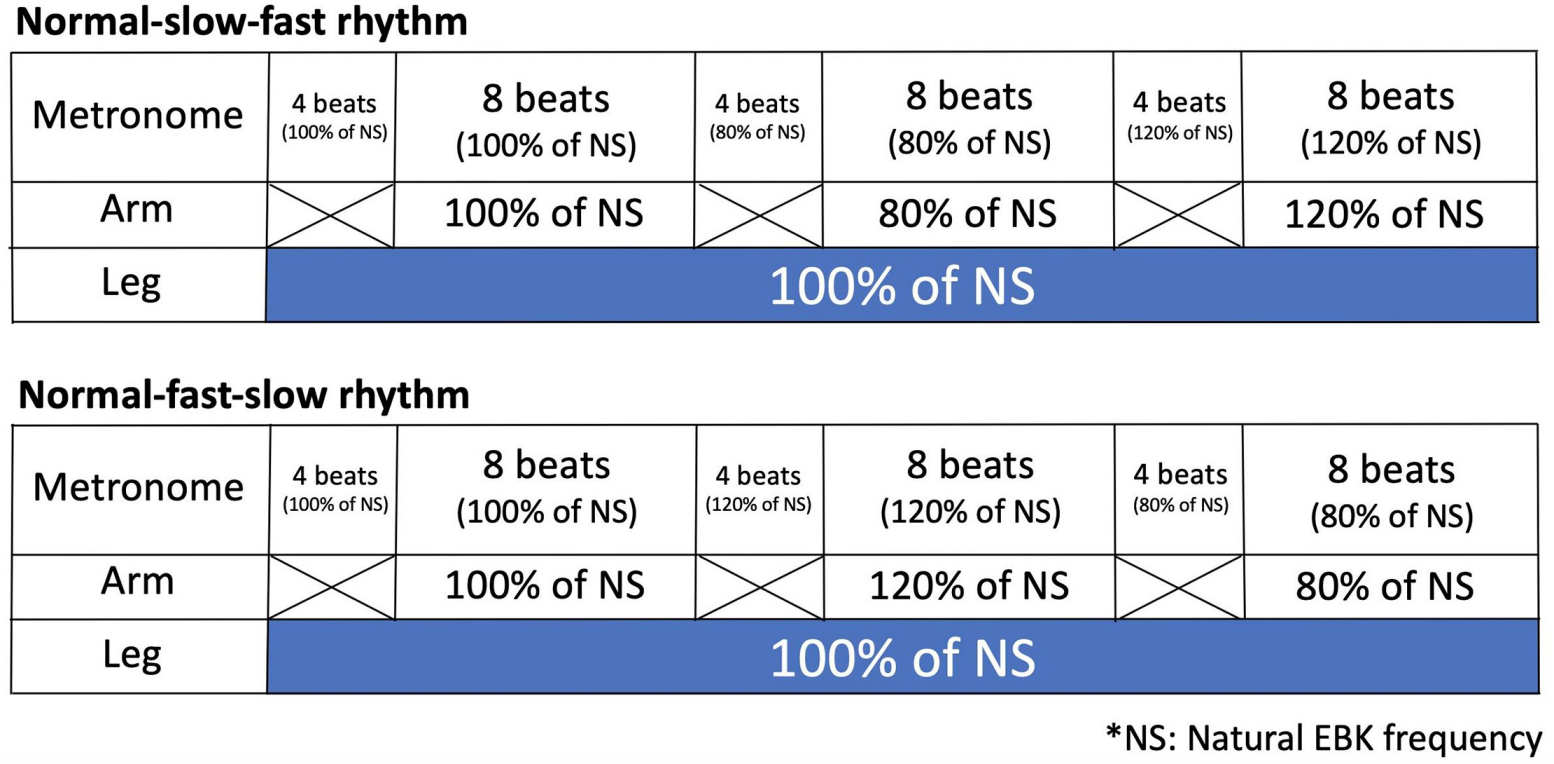
Experimental protocol for arm and leg movements in task 2, arm and leg coordination with metronome beats. * NS: Natural EBK frequency.

Fig 2 schematically summarizes the experimental setup of the present study. The participants were randomly assigned to start with either the normal-slow-fast or normal-fast-slow rhythm sequence. They were required to listen to the first four beats as a ‘ready’ cue while performing only EBKs, after which they received a signal to begin CAM in synchronization with the metronome for eight beats at each tempo. Thus, the participants performed CAMs for 24 beats in each set. Before the eight-beat slow and fast performance periods, a transition period of four beats was provided during which the participants could listen to the next tempo without making any arm movements. The participants are required to complete both sequences of rhythms three times. To minimize the effect of fatigue, they were permitted to rest as long as they considered necessary before each trial.

### Experimental procedure and data collection

**Fig 3:**
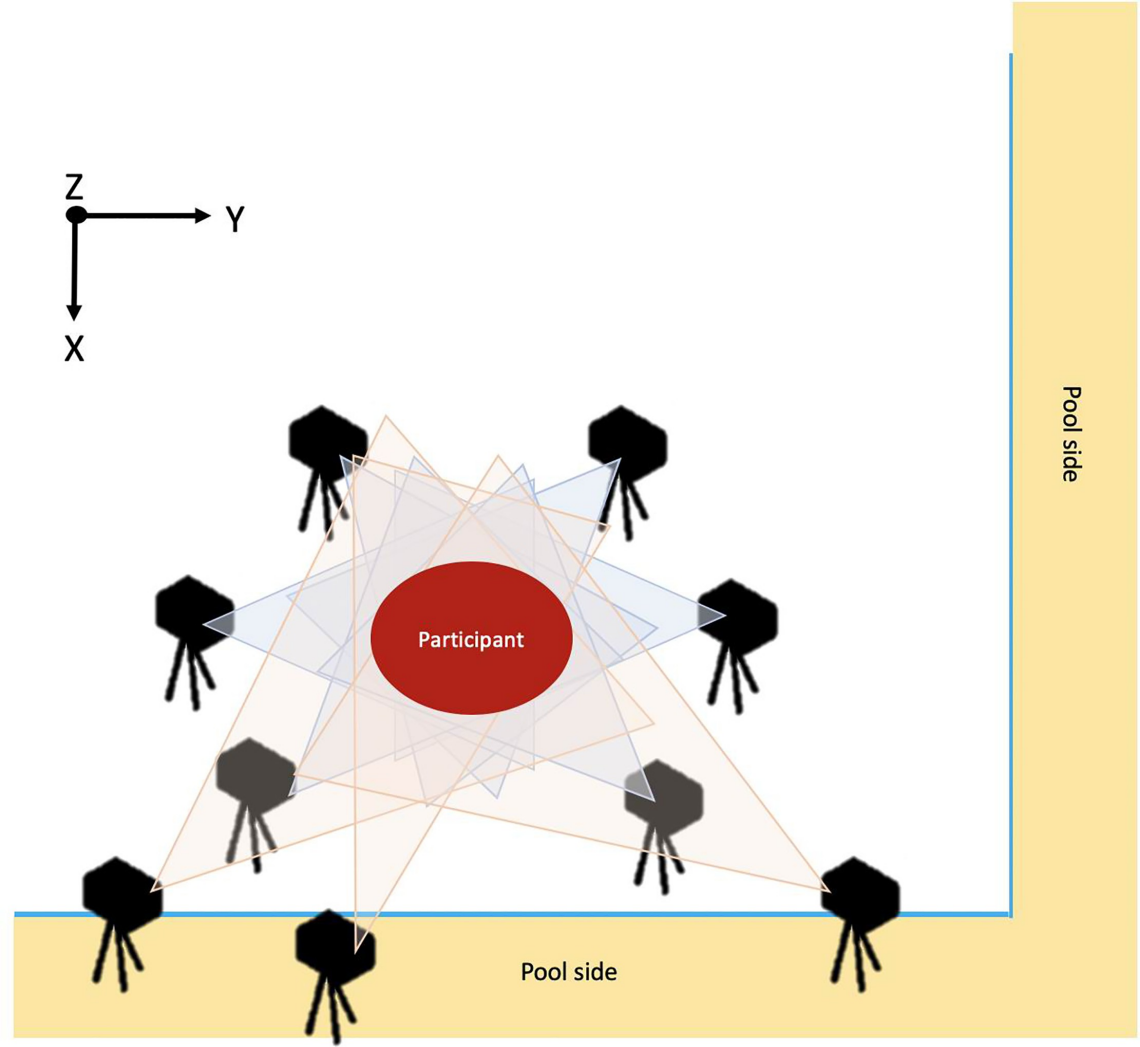
A diagram of the experimental setting.

The experiment was conducted in an indoor 50 m swimming pool. Prior to data collection, the participants performed an individual warm-up session and then had a 10 min practice session. The participants were instructed and practiced all of the tasks which they need to perform. The CAMs were checked and corrected when the participants were doing wrong movements. After that, light-emitting diode (LED) markers were attached at four anatomical landmarks (the styloid process of radius and head of ulna of the dominant hand (right and left hand for right- and left-handed athletes, respectively), xiphoid process, and lateral malleolus of the dominant leg) to quantify the motion of the wrist, ankle, and chest. A three-dimensional (3D) motion capture system (VENUS3D, Nobby Tech, Japan) consisting of six underwater cameras and three above-water cameras was situated so as to capture the areas of the body to be analyzed. A 3D direct linear transform (DLT) method using dynamic calibration was used. The standard error of underwater motion capture calibration was less than 0.3 mm. The right-handed Cartesian 3D coordinate system was used with the X-, Y- and Z-axes representing the forward, lateral and vertical directions of each participant (Fig 3). The metronome beat was created using Logic Pro X (Apple, USA.) and played through speakers. Motion data were synchronized with the sound data by means of LabVIEW (National Instruments, USA). The sampling frequency of both the motion capture system and the metronome signal was 100 Hz.

### Calculated variables and statistical analysis

Leg and arm cycles were determined from the range of motion of the ankle and wrist, respectively, along the transverse axis. The coefficient of variation (CV) of CAM range of motion was calculated to check the variability of CAM. The times at which the right ankle and wrist were extended farthest from the chest were detected using the global coordinate data collected by the motion capture system and were used to compute the duration of each cycle. The CAM and EBK frequency for each trial was calculated as the inverse of the average cycle duration of each cycle. In task 1, the average frequency over the three trials was used as the natural EBK frequency. Moreover, CV of natural EBK frequency amongst all recorded cycles was used to check the stability of EBKs in WPs and ASs.

For task 1, a two-way mixed-design analysis of variance (ANOVA) was used to compare the CV of natural EBK frequency. In this ANOVA, the three trials for each participant were treated as a within-subjects factor, while the two groups (ASs and WPs) were treated as a between-subjects factor. For task 2, a three-way mixed-design ANOVA was performed on the average frequency of CAM and EBKs between groups. Beat rates (80%, 100% and 120%) and limbs (arm and leg) were treated as within-subject factors, and the two groups (ASs and WPs) were again treated as a between-subjects factor. In addition, a two-way mixed-design ANOVA was performed on CV of CAM range of motion. Beat rates (80%, 100% and 120%) were treated as within-subject factors, and the two groups (ASs and WPs) were treated as a between-subjects factor. The Greenhouse-Geisser correction was used if the assumption of sphericity was violated. Because some factors (group and rhythm) had more than two levels, a one-way ANOVA was also performed as a post-hoc analysis if any statistically significant difference was found. All analyses were performed using IBM SPSS Statistics version 24 (IBM, NY, USA).

## Results

### Task 1: Determiningnatural EBK frequencyand its variability

**Fig 4:**
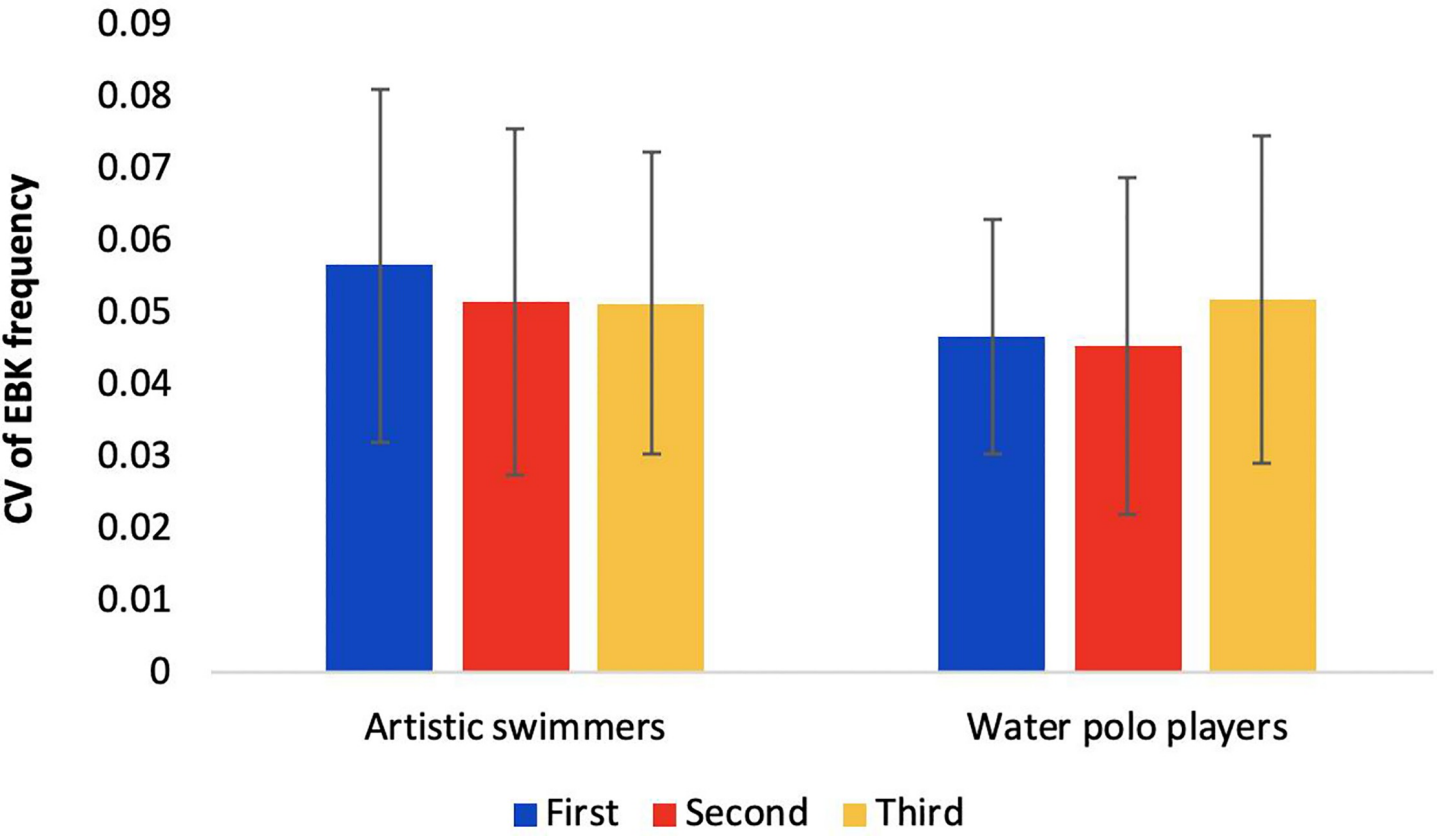
Coefficient of variation of EBK frequency over three trials for artistic swimmers and water polo players.

The average EBK frequencies amongst ASs and WPs were 82.8 ± 4.70 bpm and 74.6 ± 3.56 bpm, respectively. The results of the two-way mixed ANOVA indicate no significant main effects (*p* = .66 for the trials, *p* = .5 for the groups) or interaction between groups and trials (*F* = 1.06, *p* = .36) in the CV of EBK frequency (Fig 4).

### Task 2: Arm and leg coordination with metronome beats

The results of task 2 are reported in terms of the distribution, average of CAMs and EBKs, and CV and the typical examples of CAM range of motion. The distribution of arm and leg cycles shows the number of cycles the participants performed with each frequency during the task.

### Distribution of CAM and EBK frequencies

**Fig 5:**
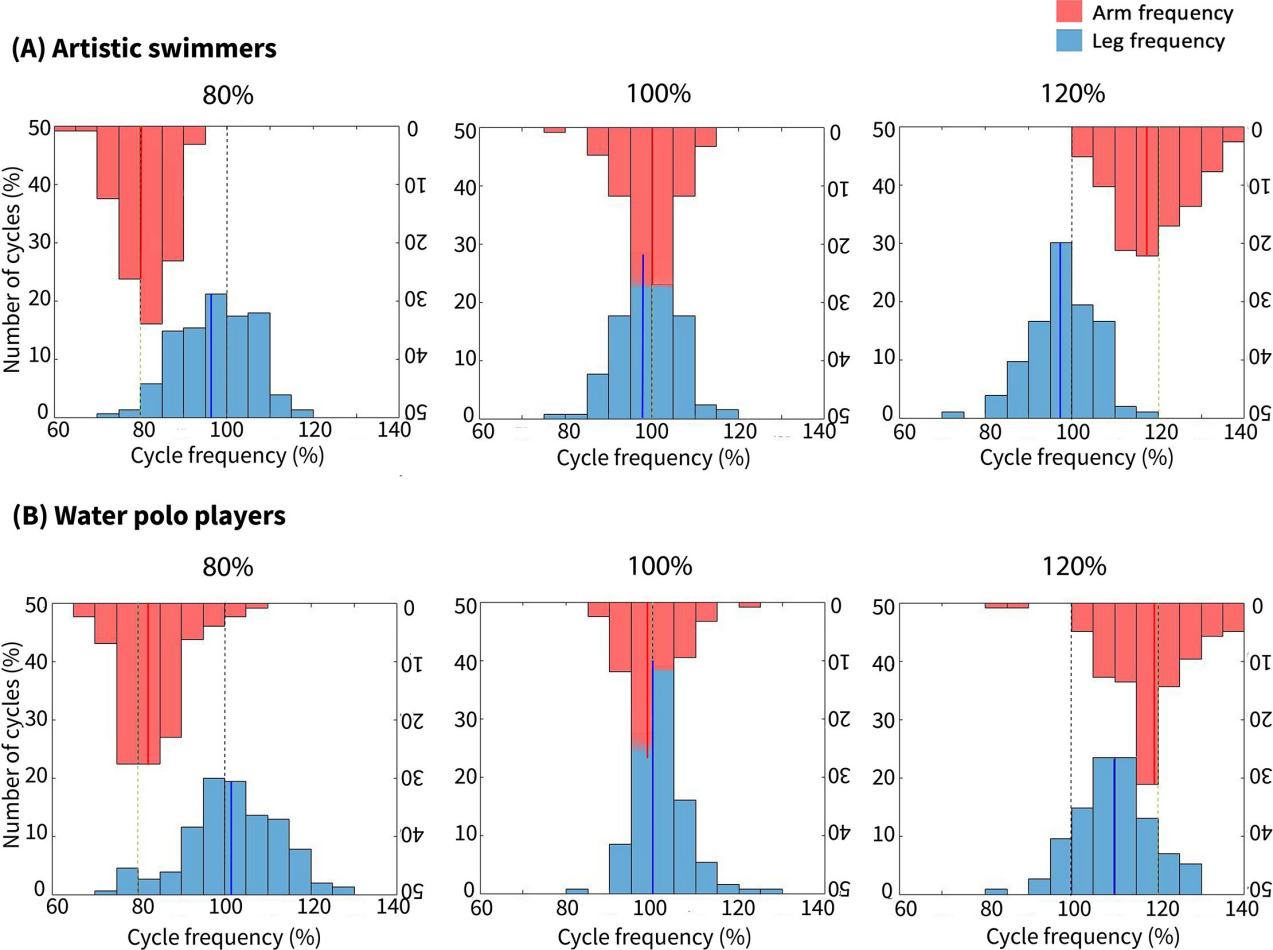
Distribution of CAM and EBK frequency in artistic swimmers (A) and water polo players (B). The horizontal axis represents cycle frequency relative to the natural EBK frequency, and the vertical axis represents the number of cycles as a percentage in relation to the total number of cycles amongst all participants. Black dotted lines represent 100% of natural EBK frequency, while green dotted lines represent the metronome beat frequency. Lastly, blue and red lines represent the mean EBK and CAM frequency, respectively.

The distributions of the CAM and EBK frequencies relative to the natural EBK frequency of ASs and WPs are displayed in Fig 5A and 5B, respectively. In all conditions, the frequency of CAMs for both ASs and WPs was distributed around the frequency of the metronome beats, as expected. For ASs, the EBK frequency was distributed around the natural EBK frequency in every condition. However, the EBK frequency for WPs was not distributed around the natural EBK frequencyin the fast condition (metronome beats at 120% of natural EBK frequency); instead, it shifted towards the tempo of the metronome beats.

### Differences in CAM and EBK frequency between ASs and WPs

**Fig 6:**
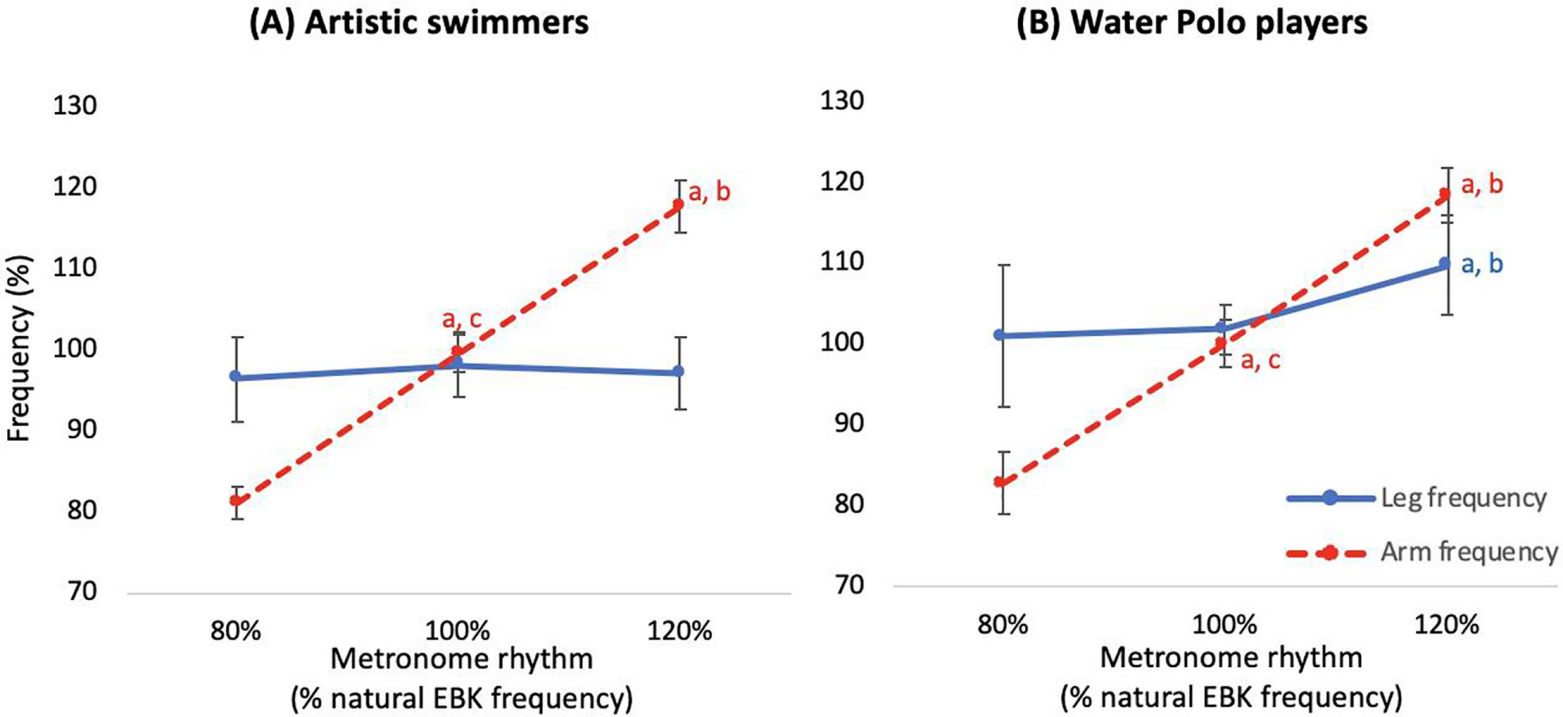
Average percentage of CAM and EBK frequencies relative to natural EBK frequency for artistic swimmers (A) and water polo players (B). a = significant difference from 80% tempo; b = significant difference from 100% tempo; c = significant difference from 120% tempo.

Fig 6 shows the average CAM and EBK frequencies by percentage relative to each individual’s natural EBK frequency. There was a significant interaction (F = 11.404, *p* < .01) amongst the three main factors: groups (ASs and WPs), limbs (arm and leg), and tasks (80%, 100%, 120%). Post-hoc analysis showed that for both groups, the CAM frequency differed significantly (*p* < .01) between the three tempos (80%, 100%, 120%). As for EBK frequency, the results showed no statistically significant difference between tempos in the AS group. In WPs, the average EBK frequency at the 120% tempo was higher than that at the 80% and 100% tempo (both *p* < .01).

### CAM range of motion in ASs and WPs

**Fig 7:**
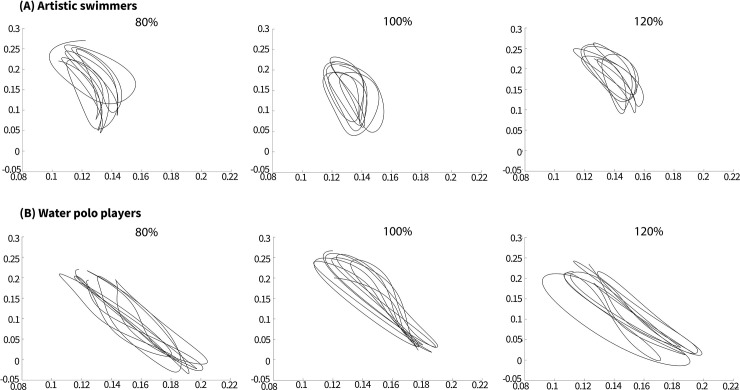
Typical examples of CAM range of motion on the Y-Z plane for artistic swimmers (A) and water polo players (B).

CV (%) of CAM range of motion in ASs and WPs were 4.17 ± 2.02 and 7.41 ± 4.69 for 80% tempo, 4.29 ± 3.61 and 5.71 ± 2.61 for 100% tempo, and 5.14 ± 4.38 and 6.06 ± 2.84 for 120% tempo, respectively. The result of two-way mixed ANOVA found no interaction between groups and tempos (*F* = 1.334, *p* = .27) and significant main effects (*p* = .54 for the tempos, *p* = .16 for the groups). Fig 7 shows typical examples of CAM.

## Discussion

This research compared two groups of athletes (ASs and WPs), one with and the other without the experience of coordinating their movements with external musical rhythms, with regard to their ability to produce entrainment and polyrhythmic movements. The results showed no statistically significant difference in the CV of natural EBK frequency between WPs and ASs, meaning that the two groups of athletes could perform EBKs in an equally stable manner when no additional tasks were involved. EBKs are a fundamental skill for both ASs and WPs [27, 28]. Each athlete has his or her own natural, preferred EBK frequency so as to optimize their energy use and provide a stable lift force during their athletic performance [26]. Without a stable EBK frequency, ASs and WPs would be unable to maintain their bodies in a stable position and would experience unnecessary loss of energy. Although the EBK is a complicated task, our study showed that ASs and WPs could perform this technique consistently at their own pace.

However, a notable difference between the groups emerged when they performed CAMs with a metronome beat in addition to EBKs. ASs could consistently perform EBKs at their natural rate even when they were also synchronizing CAMs with different metronome rhythms. In contrast, WPs were unable to perform EBKs with comparable stability when they were required to change their CAM rhythm. These results were in line with our previous research, which found that ASs had a better polyrhythmic production ability than WPs in finger- and foot-tapping tasks in different rhythms [25].

Since artistic swimming performance requires athletes to move their arms and legs at different tempos in time with the music, it is not surprising that ASs could perform limb movements skillfully at separate frequency—i.e. speeding up or slowing down their arm motions while maintaining the same EBK frequency. However, the athletic activity of WPs involves no interaction with music. Rather, when passing, shooting or defending, WPs must adopt their own limb coordination strategy to produce an effective kinetic chain that will optimize ball control [31]. Therefore, WPs are quite accustomed to coordinating their limbs with their own pace, but not with external stimuli such as a rhythmic beat. The introduction of a rhythmic beat induces synchronization of human movements with the beats [13, 15, 32, 33], and rhythmic body motions are thus modified through the interaction with the external environment, tending towards entrainment [34]. To prevent this entrainment, one limb must move in an automatic mode by utilizing central pattern generators (CPGs), which are neural circuits producing rhythmic motor movements, such as, walking and swimming, to produce rhythmic movements, whereas the other limb movements are processed through brain activities [34, 35].

Our results suggest that ASs might be more familiar with utilizing CPGs than WPs. WPs have several sensory (both visual and audio) inputs, such as ball movements, opponent movements, teammate movements, commands from teammates and coach, to process during training and competitions. To efficiently execute such motor performances requires brain process in higher motor area to control movements rather than utilizing CPGs [36]. Several studies have shown that polyrhythmic production could be more easily achieved if the participants were capable of moving one limb automatically without paying much attention to it and could thus focus on the other limb movement [37–40]. ASs could consistently maintain their polyrhythmic CAM and EBK frequencies as instructed, possibly because of their extensive artistic swimming training with music. This training would develop their ability to use CPGs to generate EBKs, enabling them to perform the EBKs with less attention and to focus on synchronizing their CAMs with the metronome beats.

According to FINA artistic swimming rules [41], ASs have a better chance of achieving a high score if they maintain good synchronization of their movements with various music rhythms and complex musical variations, especially at a fast frequency of body movements. As a result, the tempo of artistic swimming competitions has increased recently. Rodríguez-Zamora, Iglesias, Barrero, Torres-Ronda, Chaverri and Rodríguez [42] reported that the ASs in their study had trained repeatedly to move in synchronization with their teammates and the music for more than 40 hours per week. Therefore, they were more expert in coordinating their limb movements with music than WPs, especially with a fast music tempo, allowing them to experience less entrainment than WPs.

Interestingly, our results showed that entrainment did not tend to occur when WPs performed CAMs at 80% of natural EBK frequency, suggesting that not every interaction pattern between limb motion and external environmental stimuli tends equally towards entrainment. This result might be related to a technical aspect of EBKs that is required to maintain body height. Since there is a positive relationship between vertical force and eggbeater kick velocity (i.e. frequency) [43], WPs might not have been able to slow down their EBKs even when performing CAMs at a slower rate, because reducing their EBK frequency would cause difficulty in maintaining their body height. As seen from Fig 5, there were some cycles performed at 80% (or less) of natural EBK frequency. However, these cycles were only accounted for 5% of the total cycle number. Therefore, it is likely that performing eggbeater kicks lower than 80% of natural EBK frequency is not feasible to keep the required body height (upper edge of the sternum).

Although our results indicate the benefit of experience in coordinating one’s movements with music as a way to produce polyrhythmic movements between upper and lower extremities, some limitations of this study should be noted. First, the groups in our experiment were of opposite genders, because artistic swimming is competed primarily by females and water polo is more popular amongst males. However, given that gender has little effect on polyrhythmic production ability, as reported by Aoki, Furuya and Kinoshita [44], it is likely that the differences observed in this study reflected the two athlete groups’ differing backgrounds in limb coordination with music rather than gender differences. Nevertheless, more research on training in arm–leg coordination and its relationship to music exposure will help us to understand more fully the benefits of musical interaction in facilitating such coordination in athletes.

Our findings emphasized the benefit of experiences in coordinating movements with music to polyrhythmic productions. Music has been suggested to be useful for, and an integral part of, polyrhythmic production [45]. Music and dancing lessons that require movement coordination under different tempos/rhythms probably benefit ASs by improving their polyrhythmic production skills. Artistic swimming coaches can, therefore, benefit from including music and dancing lessons as a part of training sessions. Potentially, checking musical or dancing experiences of athletes would also be beneficial for talent identification of ASs. Moreover, WPs could also benefit from training with music or external sound rhythm in improving their polyrhythmic production skills. Training to produce polyrhythmic movements skillfully would allow WPs to change their upper body movements with little changes in EBK frequency, which would potentially make their movements more stable and efficient.

## Conclusion

In this study, we investigated polyrhythmic production ability and entrainment between two groups of athletes—ASs and WPs—who had different experiences in coordinating their movements with music. We found that WPs and ASs had a similar CV of natural EBK frequency, implying that both groups could perform EBKs in a stable manner when no additional tasks were required of them. When the participants were asked to perform polyrhythmic production of CAMs and EBKs, ASs maintained a consistent EBK rate while also completing CAMs in synchronization with a variety of metronome rhythms. However, WPs were unable to perform EBKs at their natural rate while also performing CAMs at a fast tempo. The results indicate that ASs are more capable of generating EBKs consistently regardless of the external rhythmic stimulus.

## Supporting information

S1 Table Eggbeater kick frequency over three trials of artistic swimmers and water polo players in task 1. (PDF)

S2 Table Eggbeater kick and circular arm movement frequency in task 2: Normal-slow-fast task. (PDF)

S3 Table Eggbeater kick and circular arm movement frequency in task 2: Normal-fast-slow task. (PDF)

S4 Table Coefficient of variation of circular arm movement range of motion. (PDF)
