# Supplementary material for: The effect of experience in movement coordination with music on polyrhythmic production: Comparison between artistic swimmers and water polo players during eggbeater kick performance
Source: PLoS One. 2020 Aug 25;15(8):e0238197. doi: 10.1371/journal.pone.0238197 (PMC7447008; doi:10.1371/journal.pone.0238197)
Supplement: S1 Table — (PDF) [file pone.0238197.s001.pdf]

S1 Table. Eggbeater kick frequency over three trials of artistic swimmers and water polo players in task1

Participant	Artistic swimmers				Water polo players			
	Trial	Mean	SD	Average over three trials	Trial	Mean	SD	Average over three trials
**1**	1	68.10	2.20	69.6	1	74.45	3.67	75.73
	2	70.05	3.27		2	76.51	5.26	
	3	70.65	2.97		3	76.22	4.824	
**2**	1	86.25	5.63	89.48	1	70.23	3.55	69.64
	2	92.49	2.52		2	70.00	1.73	
	3	89.71	3.11		3	68.71	2.27	
**3**	1	86.94	2.18	84.92	1	82.66	4.60	83.28
	2	85.37	2.40		2	83.50	3.01	
	3	82.45	3.67		3	83.68	6.19	
**4**	1	83.82	2.74	85.49	1	95.75	7.57	93.06
	2	86.13	2.28		2	90.86	8.54	
	3	86.54	1.79		3	92.57	8.81	
**5**	1	76.49	4.81	76.43	1	72.49	1.99	71.91
	2	75.46	5.37		2	71.72	1.72	
	3	77.34	4.12		3	71.54	2.26	
**6**	1	90.64	8.20	87.1	1	62.58	2.42	62.14
	2	85.09	7.23		2	62.46	2.06	
	3	85.56	7.45		3	61.37	2.06	
**7**	1	87.20	6.70	86.65	1	68.65	2.75	70.67
	2	84.86	7.00		2	71.79	2.20	
	3	87.89	14.92		3	71.56	3.17	
**8**	1	81.53	6.60	78.87	1	80.44	1.97	83
	2	78.81	5.05		2	83.37	2.89	
	3	76.28	4.35		3	85.18	2.60	
**9**	1	86.42	3.24	86.43	1	58.84	2.52	57.96
	2	86.88	3.27		2	57.14	2.85	
	3	85.99	3.69		3	57.91	2.52	
**Mean**			4.70	82.78			3.56	74.15
