# Supplementary material for: The effect of experience in movement coordination with music on polyrhythmic production: Comparison between artistic swimmers and water polo players during eggbeater kick performance
Source: PLoS One. 2020 Aug 25;15(8):e0238197. doi: 10.1371/journal.pone.0238197 (PMC7447008; doi:10.1371/journal.pone.0238197)
Supplement: S2 Table — (PDF) [file pone.0238197.s002.pdf]

S2 Table. Eggbeater kick and circular arm movement frequency in task2: Normal-slow-fast task

Participant	Artistic swimmers						Water polo players					
	Eggbeater kick frequency			Circular arm movement frequency			Eggbeater kick frequency			Circular arm movement frequency		
	100% of NS	80% of NS	120% of NS	100% of NS	80% of NS	120% of NS	100% of NS	80% of NS	120% of NS	100% of NS	80% of NS	120% of NS
1	100.20	99.11	97.53	100.24	79.75	116.55	107.46	107.08	118.02	100.58	93.11	122.70
2	93.35	86.18	97.22	97.61	78.10	110.48	100.43	97.38	102.78	103.45	85.55	124.73
3	103.24	103.66	105.78	102.06	80.86	117.95	101.13	88.37	105.95	107.47	85.90	121.73
4	98.11	99.05	98.56	95.94	84.04	114.94	101.50	108.47	109.77	96.04	78.73	116.01
5	97.38	94.82	93.74	105.28	82.46	119.42	103.89	101.72	111.28	99.31	82.87	112.60
6	99.30	101.64	99.44	101.55	82.11	117.96	99.55	87.96	102.10	98.08	78.74	118.71
7	96.16	92.25	92.44	97.78	79.11	111.05	100.67	98.53	106.66	101.40	86.94	122.49
8	98.71	93.17	97.98	99.36	85.94	120.39	100.20	97.33	106.69	101.16	81.58	119.65
**9**	98.90	97.43	96.78	99.48	78.95	120.90	104.44	107.82	118.73	98.60	78.47	116.91
Mean	98.37	96.37	97.72	99.92	81.26	116.63	102.14	99.41	109.11	100.68	83.54	119.50
SD	2.73	5.36	3.77	2.79	2.60	3.80	2.59	7.74	6.00	3.34	4.86	3.85
