# Supplementary material for: The effect of experience in movement coordination with music on polyrhythmic production: Comparison between artistic swimmers and water polo players during eggbeater kick performance
Source: PLoS One. 2020 Aug 25;15(8):e0238197. doi: 10.1371/journal.pone.0238197 (PMC7447008; doi:10.1371/journal.pone.0238197)
Supplement: S3 Table — (PDF) [file pone.0238197.s003.pdf]

S3 Table. Eggbeater kick and circular arm movement frequency in task2: Normal-fast-slow task

Participant	Artistic swimmers						Water polo players					
	Eggbeater kick frequency			Circular arm movement frequency			Eggbeater kick frequency			Circular arm movement frequency		
	100% of NS	120% of NS	80% of NS	100% of NS	120% of NS	80% of NS	100% of NS	120% of NS	80% of NS	100% of NS	120% of NS	80% of NS
1	95.17	94.80	98.02	99.97	117.78	80.92	110.40	119.48	110.08	104.41	119.94	88.46
2	97.30	95.37	96.76	97.61	116.64	79.43	101.33	99.23	98.20	98.56	113.00	80.34
3	103.62	104.18	104.75	103.42	119.88	80.89	98.83	112.56	81.76	98.28	114.34	83.11
4	101.85	101.33	101.09	97.59	116.64	81.79	100.69	112.63	105.65	97.72	122.11	79.19
5	102.90	99.34	96.23	100.84	122.92	82.20	99.69	108.67	109.66	101.58	115.41	82.00
6	90.20	90.65	89.47	96.76	121.64	78.36	102.39	108.82	99.26	97.13	117.66	82.13
7	90.45	87.84	93.94	98.17	113.91	80.46	103.40	109.76	100.82	96.33	119.64	79.69
8	100.07	94.49	89.31	99.09	121.18	80.91	98.75	104.07	102.58	101.10	116.45	82.84
**9**	100.65	98.96	99.93	100.17	119.79	82.97	98.67	119.55	116.24	98.81	117.48	80.22
Mean	98.02	96.33	96.61	99.29	118.93	80.88	101.57	110.53	102.69	99.32	117.34	82.00
SD	5.10	5.17	5.13	2.07	2.89	1.39	3.71	6.58	9.80	2.56	2.90	2.80
