# Supplementary material for: The effect of experience in movement coordination with music on polyrhythmic production: Comparison between artistic swimmers and water polo players during eggbeater kick performance
Source: PLoS One. 2020 Aug 25;15(8):e0238197. doi: 10.1371/journal.pone.0238197 (PMC7447008; doi:10.1371/journal.pone.0238197)
Supplement: S4 Table — (PDF) [file pone.0238197.s004.pdf]

S4 Table. Coefficient of variation of circular arm movement range of motion

	Artistic swimmers			Water polo players		
	80% of NS	100% of NS	120% of NS	80% of NS	100% of NS	120% of NS
1	5.03	2.68	4.66	5.03	4.92	5.41
2	3.31	1.59	2.97	3.31	6.29	3.87
3	12.61	13.03	15.34	12.61	3.59	4.13
4	6.21	4.69	7.68	6.21	5.16	5.87
5	17.58	6.34	6.83	17.58	11.47	11.43
6	5.18	1.75	2.36	5.18	3.63	10.40
7	6.77	3.43	1.61	6.77	4.73	4.98
8	6.54	2.67	2.27	6.54	8.05	4.49
**9**	5.03	2.41	2.53	5.03	4.92	5.41
Mean	7.41	4.29	5.14	7.41	5.71	6.06
SD	4.69	3.61	4.38	4.69	2.61	2.84
